# Noninvasive monitoring of inspiratory effort in mechanical ventilation: a dual-database bibliometric analysis from 1990 to 2025

**DOI:** 10.3389/fmed.2025.1747437

**Published:** 2026-01-12

**Authors:** Xu An, Dan Hou, Ming-Yue Miao, Yi-Min Zhou, Saiping Qi, Linlin Zhang, Hongliang Li, Jian-Xin Zhou

**Affiliations:** 1 Department of Critical Care Medicine, Emergency and Critical Care Medical Center, Beijing Shijitan Hospital, Capital Medical University, Beijing, China; 2 Clinical and Research Center on Acute Lung Injury, Capital Medical University, Beijing, China; 3 Department of Critical Care Medicine, Beijing Tiantan Hospital, Capital Medical University, Beijing, China; 4 Department of Neurocritical Care, Beijing Anzhen Hospital, Capital Medical University, Beijing, China

**Keywords:** airway occlusion pressure, ARDS, bibliometrics, inspiratory effort, lung and diaphragm-protective ventilation, lung-protective ventilation, mechanical ventilation

## Abstract

**Introduction:**

This study conducts a bibliometric analysis to map the intellectual structure, evolution, and emerging trends in research on airway pressure-based indexes for monitoring inspiratory effort.

**Methods:**

Systematic searches of the Web of Science Core Collection (WOSCC) and Pubmed were performed for publications dated between 1990 and 2025. Bibliometric parameters, including publication trends, country and affiliation contributions, author influence, journal distribution, keyword co-occurrence, and reference co-citation networks, were analyzed using Bibliometrix and CiteSpace.

**Results:**

The analysis included 291 publications from WOSCC. The annual publication output showed a near U-shaped trend, with an initial decline after the 1990s, followed by a strong resurgence after 2011. Italy was the most productive country, followed by the USA and France. The Institut National de la Sante et de la Recherche Medicale emerged as the leading institution. The journal Chest published the most articles, while the American Journal of Respiratory and Critical Care Medicine had the highest total citations. Laurent Brochard was identified as the most prolific and influential author. Keyword analysis highlighted “occlusion pressure” and “mechanical ventilation” as core themes. Reference co-citation clustering revealed major research domains, including “acute respiratory distress syndrome,” “self-inflicted lung injury,” and “nasal high flow.” Burst detection analysis indicated that “respiratory drive,” “lung injury,” and “critically ill patients” are emerging research frontiers. Complementary analysis of 242 PubMed clinical studies confirmed these trends and highlighted growing clinical focus on “fluid responsiveness” and “amyotrophic lateral sclerosis.”

**Conclusion:**

Research on airway pressure-based indices has evolved from physiological studies into a crucial clinical tool for respiratory monitoring. The field exhibits strong international collaboration and emphasizes core areas, including acute respiratory failure and lung-protective ventilation. Analysis of clinical study data confirms these trends and highlights emerging applications in the assessment of fluid responsiveness and neuromuscular disorders. These findings support the ongoing development of personalized ventilation strategies based on monitoring respiratory effort.

## 1 Introduction

Maintaining inspiratory effort within a physiological range is a key therapeutic target in mechanically ventilated patients. Insufficient inspiratory effort is associated with ventilator-induced diaphragmatic atrophy and dysfunction (1, 2), while excessive inspiratory effort can exacerbate lung stress and strain, leading to patient self-inflicted lung injury (3, 4). Both insufficient and excessive inspiratory efforts may contribute to patient-ventilator asynchrony and compromise hemodynamic monitoring, ultimately resulting in adverse clinical outcomes (5, 6). Typically, inspiratory drive and effort are synchronized, which reflects the relationship between the intensity of the respiratory drive and resulting muscle contraction (7). However, in critically ill patients, this synchronization can be disrupted by factors such as respiratory muscle dysfunction and ventilator settings (8, 9).

Accurately monitoring inspiratory effort has become central to advancing the paradigm of lung-protective ventilation (10). The critical importance of this paradigm is particularly evident in the management of Acute Respiratory Distress Syndrome (ARDS), where the primary therapeutic goal is to mitigate ventilator-induced lung injury while maintaining adequate gas exchange (11). Traditional lung-protective ventilation emphasizes limiting tidal volume and plateau pressure; however, a growing body of evidence underscores that dysregulated inspiratory effort—whether insufficient or excessive—can independently undermine these protective goals (11, 12). Excessive effort can lead to high transpulmonary pressure and promote patient P-SILI, even when low tidal volumes are employed (3, 4, 12). Conversely, insufficient effort contributes to diaphragmatic dysfunction and atrophy, potentially delaying weaning (2). Therefore, integrating the monitoring and titration of inspiratory effort is now recognized as an essential component of a comprehensive lung-protective strategy, which is unequivocally associated with improved survival in ARDS (13). The pursuit of this refined approach directly motivates the need for practical, non-invasive methods for assessing inspiratory effort at the bedside.

Diaphragmatic electrical activity and esophageal manometry are regarded as reference techniques for assessing inspiratory drive and effort; however, their applications are limited by several limitations such as invasiveness, calibration requirements, and the need for specialized expertise in interpretation and data acquisition (14–17). Alternatively, airway pressure-based indexes (e.g., airway occlusion pressure at 100 ms (P0.1), whole-breath occlusion pressure (ΔPocc), and the pressure-muscle index (PMI)), have been proposed as non-invasive surrogates to estimate inspiratory effort (7, 18). These parameters can be easily available on most modern ventilators (19–22). P0.1 reflects output from the brainstem’s respiratory centers (19). Although primarily a measure of respiratory drive, P0.1 is also a reliable surrogate for inspiratory effort in most patients (23). ΔPocc, assessed during an expiratory occlusion, is defined as the maximum decrease in airway pressure during an entire occluded breath (18). PMI is calculated as the difference between plateau pressure and peak inspiratory pressure and can discriminate between high and low levels of inspiratory effort during pressure support ventilation (18, 24). Currently, these airway pressure-based indexes are practical tools for the bedside assessment of inspiratory effort (18).

Bibliometric analysis provides an effective approach for quantifying overarching trends in research activity and elucidating collaborations among relevant institutions (25, 26). This method can evaluate the volume and temporal evolution of scientific output across countries and years within major domains (27). Furthermore, through information visualization and quantitative mapping, bibliometrics can reveal dynamic shifts and identify research hotspots in specific fields (27, 28). Therefore, the bibliometric analysis of airway pressure-based indexes for monitoring inspiratory effort may reveal valuable insights. Scientific mapping provides an effective means of bibliometric visualization and clarifies the current state and developmental trends. Accordingly, this study aimed to achieve the following objectives: (1) describe the current research landscape of airway pressure-based indexes for monitoring inspiratory effort by analyzing publication trends and contributions across countries, affiliations, journals and authors; (2) examine collaborative networks; (3) identify major research themes and hotspots through keyword co-occurrence and reference co-citation analysis; and (4) highlight emerging frontiers and potential future directions in the field.

## 2 Materials and methods

### 2.1 Data source and collection

We selected the Science Citation Index Expanded of the Web of Science Core Collection (WOSCC) as the scope of bibliometric analysis. Data collection was conducted on a single day, November 10, 2025, to avoid bias caused by frequent database renewal. The literature from 1985 to 2025 was retrieved, and search terms were as follows: “(TS = (“P0.1” OR “P100” OR “P-0.1” OR “P(0.1)” OR “occlusion pressure*” OR “PMI” OR “pressure muscle index” OR “POCC” OR “occluded inspiratory airway pressure*” OR “occluded expiratory airway pressure swing” OR “airway pressure swing during occlusion”)) AND (TS = (“inspiratory effort*” OR “breathing effort*” OR “breath effort*” OR “respiratory effort*” OR “respiratory muscle effort*” OR “inspiratory muscle effort*” OR “inspiratory reserve volume*” OR “work of breathing” OR “WOB” OR “breathing work*” OR “work of inspiratory” OR “inspiratory work*” OR “work of respiratory” OR “respiratory work*” OR “respiratory capacity” OR “inspiratory capacity” OR “neuromuscular drive*” OR “respiratory drive*” OR “inspiratory drive*“OR “respiration drive*” OR “inspiration drive” OR “breathing drive” OR “breath drive”)).”

The PubMed search strategy is described in the Supplementary material. To assess the reproducibility of keyword trends identified in the WOSCC within a medically oriented database, we evaluated clinical study data from PubMed. WOSCC was selected as the primary source for the overarching bibliometric analysis due to its extensive disciplinary scope and diversity of document types. It was supplemented by PubMed, a specialized clinical repository, to validate trends found in trial-focused literature. This dual-database approach allows the project to capture both the broad spectrum of research activities through WOSCC and the focused depth of clinical evidence via PubMed (29). Notably, the two databases were not merged into a single deduplicated corpus; instead, they were analyzed in parallel to compare and contrast research patterns between a broad scientific repository and a specialized clinical database.

All the data was available from online databases, and ethics committee approval or informed consent was not required.

### 2.2 Statistical analysis

First, a preliminary analysis of the annual output of the literature was carried out using the search results from the WOSCC. The Biblimetrix software package in R (version 4.3.1) and Citespace (version 6.4.R1) was used to analyze all literature. The Biblioshiny web interface was utilized for basic analyses, including the distribution of publications by country, affiliation, journal and author. CiteSpace was employed for advanced network visualization and analysis. The parameters were configured as follows: the time span was set from 1990 to 2025 with one-year time slices; for the node type “Country,” the selection criteria were set to g-index (k = 25) and Top N (*N* = 50); for “Author,” the criteria included g-index (k = 100) and Top N (*N* = 50); and for “Keyword,” g-index (k = 25) and Top N (*N* = 50) were applied. Visual network analyses were conducted for countries, authors, and keywords. Additionally, burst detection analysis was performed to identify emerging keywords and highly cited references. These complementary approaches provided a comprehensive quantitative and visual exploration of the research landscape.

## 3 Results

### 3.1 Publication outputs

**Figure 1:**
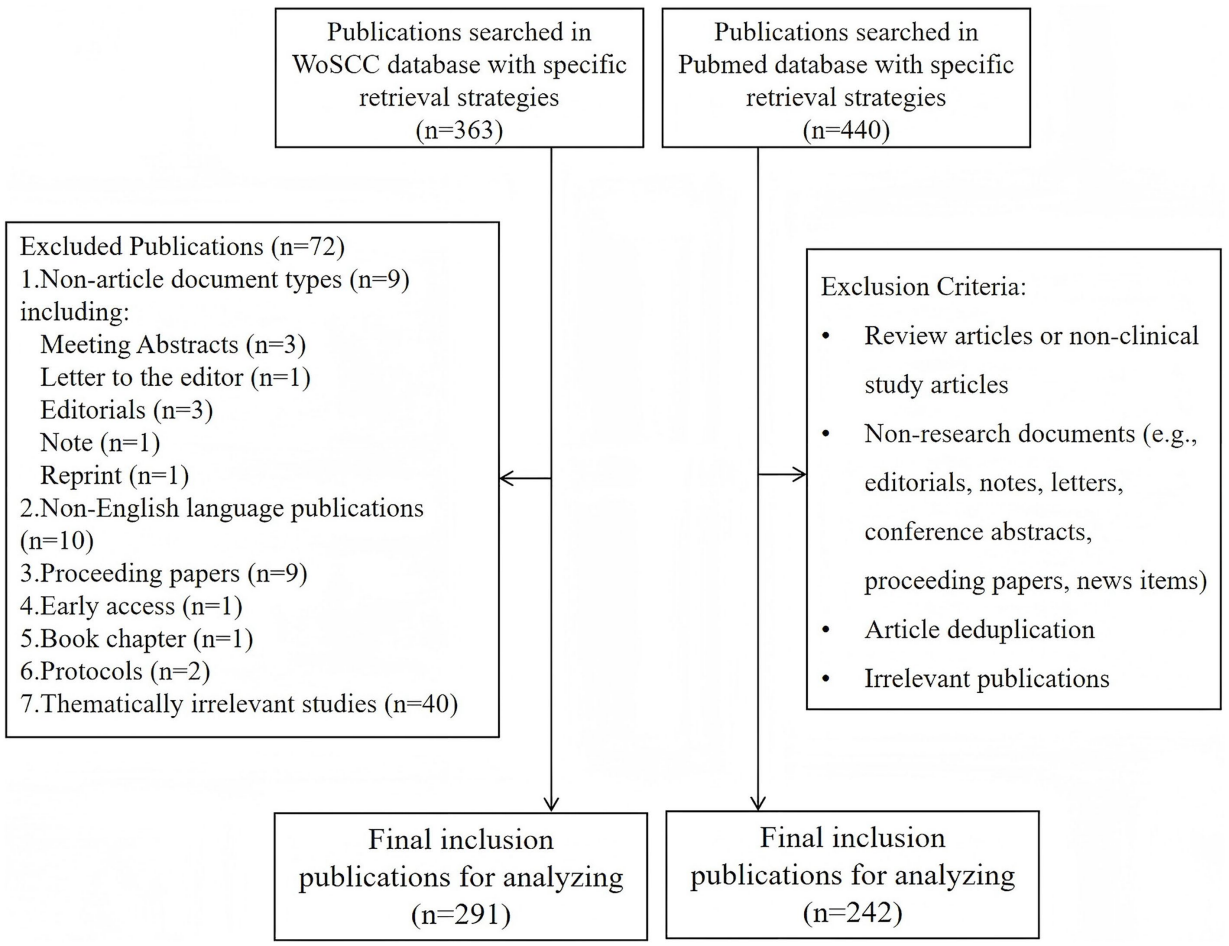
Flowchart of publications including and excluding. WoSCC, Web of Science Core Collection.

**Figure 2:**
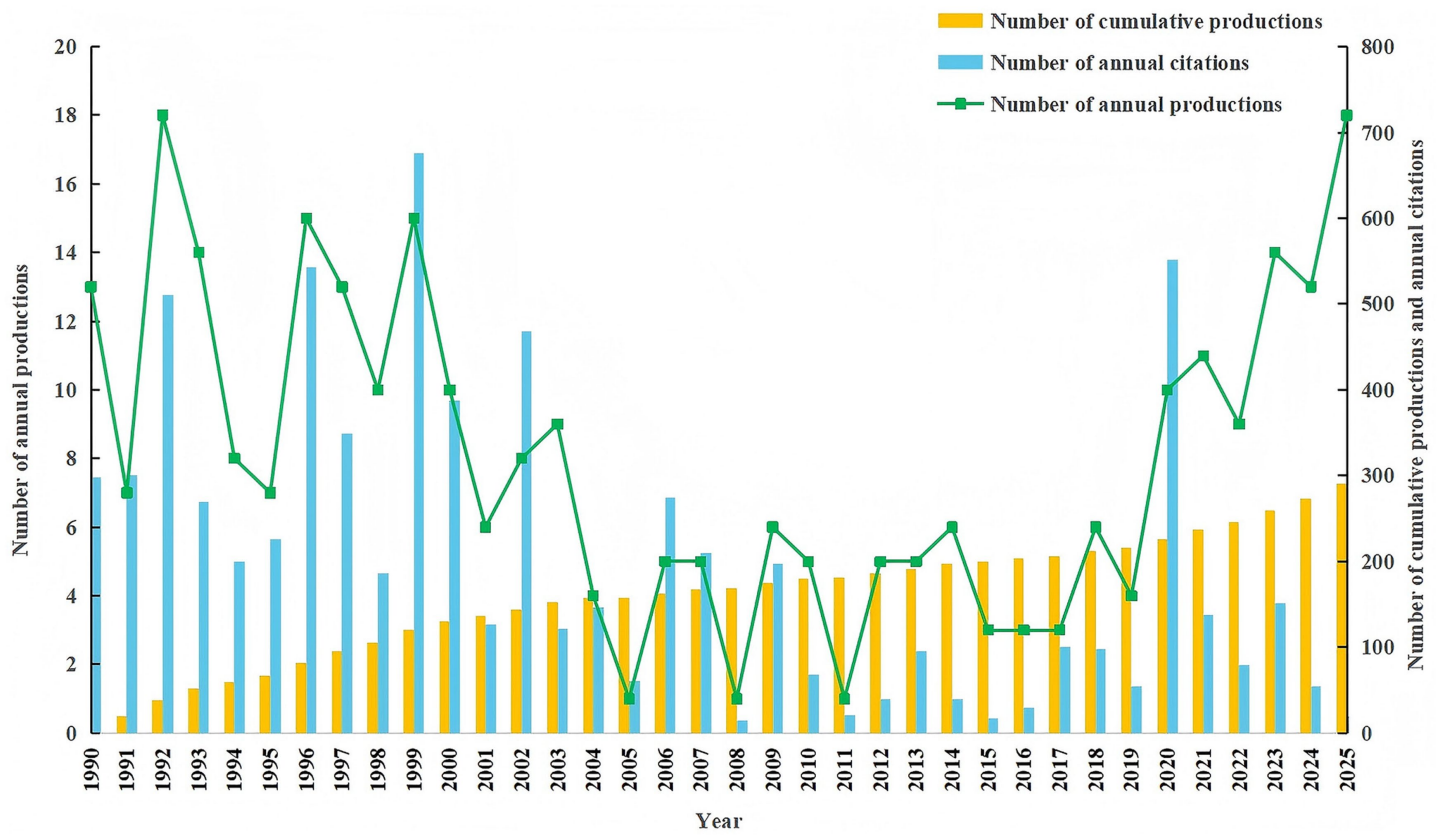
Annual publications, cumulative publications, and annual citations in the WOSCC between 1990 and 2025.

The search strategy of the WOSCC identified 291 eligible publications spanning a 35-year period (1990–2025), with 271 (93.13%) original articles and 20 (6.87%) reviews. The flow diagram is presented in Figure 1. The annual publication output is presented in Figure 2. The annual publication output exhibits a near U-shaped trend over the study period. Initially, output was high in the early 1990s, followed by a decline reaching its lowest point around 2008–2011. Subsequently, a steady recovery is observed, with annual outputs surpassing earlier levels in recent years. Annual citation counts fluctuated during this timeline, with notable peaks in 1999 and 2020, indicating periods of heightened scholarly impact. This overall trend in publication output reflects a resurgence of research activity and interest in the field, underscoring its dynamic changes in scholarly engagement over time. It is important to note that since data collection was completed on November 10, 2025, the publication and citation count for 2025 are incomplete and do not reflect the full year.

### 3.2 Contribution of countries and affiliations

**Figure 3:**
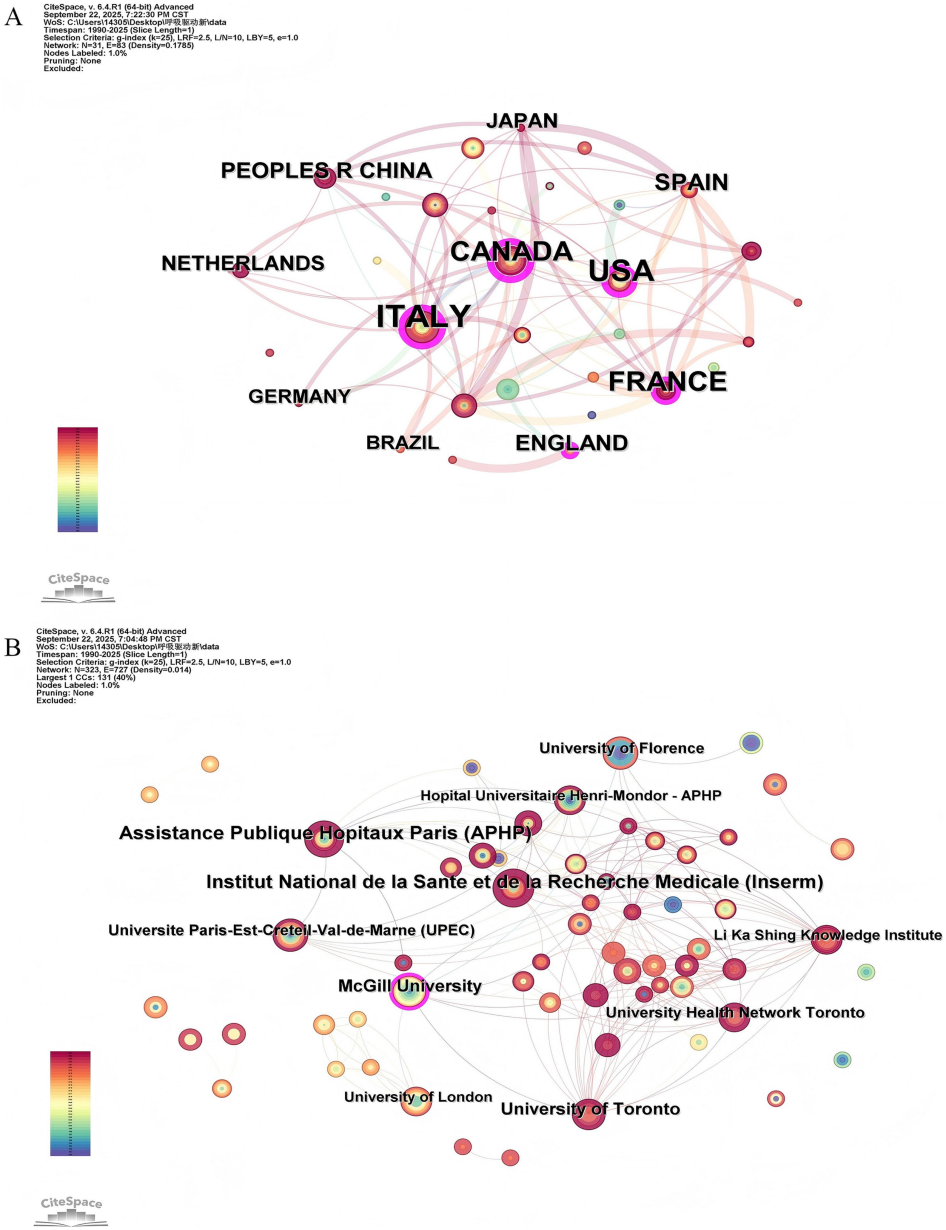
**(A)** Visualization map of the scientific collaboration network analysis among countries/regions. **(B)** Visualization map of the scientific collaboration network analysis among affiliations, with the threshold set to a minimum of 3 publications per affiliation.

Between 1990 and 2025, 32 countries or regions and 356 institutions contributed to publications on airway pressure-based indexes for monitoring inspiratory effort. As shown in Table 1, the publications were widely distributed across countries/regions and affiliations. According to a whole counting method, where each country/region on a publication is credited, Italy produced the most documents (*n* = 63), followed by the USA (*n* = 54) and France (*n* = 38) At the institutional level, the Institut National de la Sante et de la Recherche Medicale published the most articles (*n* = 19), followed by Assistance Publique Hopitaux Paris (*n* = 18) and the University of Toronto and McGill University (each with *n* = 13). Figures 3A,B illustrate active collaboration among different countries and institutions. Canada was the most active country in international cooperation, while the Institut National de la Sante et de la Recherche Medicale was the most collaborative institution. These findings reflect strong and diverse international engagement in this research area.

**Table 1:** The top 10 countries/regions and affiliations contributing to publications.

Ranks	Country/region	Frequency	Affiliation	Frequency
1	Italy	63	Institut National de la Sante et de la Recherche Medicale	19
2	USA	54	Assistance Publique Hopitaux Paris	18
3	France	38	University of Toronto	13
4	Canada	37	McGill University	13
5	Spain	25	University Health Network Toronto	10
6	England	16	Universite Paris-Est-Creteil-Val-de-Marne	9
7	China	15	University of Florence	9
8	Germany	14	University of London	8
9	Japan	14	Li Ka Shing Knowledge Institute	8
10	Netherlands	14	Hopital Universitaire Henri-Mondor	8

### 3.3 Analysis of journal output and author contributions

Until November 10, 2025, there were 291 publications on airway pressure-based indexes for monitoring inspiratory effort published across 94 journals, with 8 journals having at least 10 publications each. The top 10 high-yielding journals are listed in Table 2. These journals are predominantly ranked in the JCR Q1 quartile, indicating high-quality publication outlets. Among them, Chest published the most articles (*n* = 28), while the American Journal of Respiratory and Critical Care Medicine accumulated the highest total citations (TC) (TC = 1,009), reflecting strong academic influence. Journals such as the European Respiratory Journal and Intensive Care Medicine also demonstrated notable impact with high citation counts (TC = 670 and TC = 634, respectively). The top 10 most highly cited publications are listed in Supplementary Table S1.

**Table 2:** The top 10 high-yielding journals in research.

Ranks	Sources	Articles	Total citations	H-index	IF (2024)	JCR (2024)
1	Chest	28	828	17	8.60	Q1
2	European Respiratory Journal	17	670	12	21.00	Q1
3	Respiratory Care	16	174	8	2.10	Q2
4	American Journal of Respiratory and Critical Care Medicine	13	1,009	12	19.40	Q1
5	Critical Care	12	233	6	9.30	Q1
6	Critical Care Medicine	12	389	11	6.00	Q1
7	Intensive Care Medicine	12	634	11	21.20	Q1
8	Annals of Intensive Care	10	209	8	5.50	Q1
9	Journal of Applied Physiology	9	164	8	3.30	Q1
10	Respiration Physiology	9	137	6	–	–

H-index, Hirsch index; IF, impact factor; JCR, journal citation reports.

**Figure 4:**
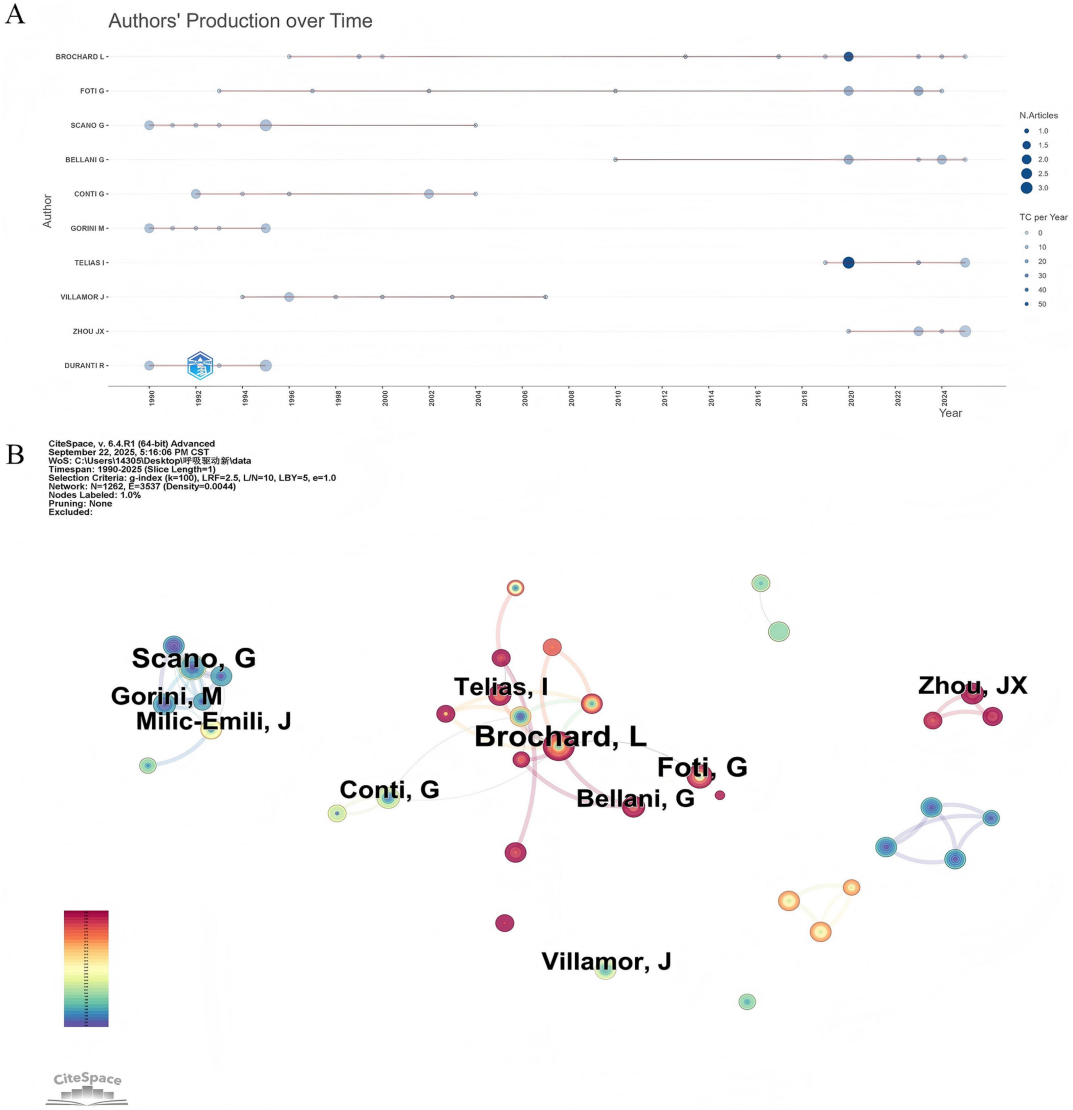
Analysis of author contributions. **(A)** Authors’ production over time. **(B)** Visualization map of the scientific collaboration network analysis among authors, with the threshold set to a minimum of 3 publications per author.

The top 10 most productive authors are listed in Table 3. Laurent Brochard from the University of Toronto, Canada, was the leading author with 11 publications, achieving the highest TC (TC = 704) and a Hirsch index (H-index = 9). Notably, six of the authors were from Italy, indicating a strong research focus in this country. The publication and citation metrics of these authors reflect their substantial impact in this field. Analysis of annual publication citation trends among these highly productive authors revealed distinct chronological phases (Figure 4A). Scano G, Gorini M and Duranti R were among the earlier contributors, starting in 1990. Foti G and Brochard L began sustained research efforts in 1993 and 1996, respectively, and maintained consistent academic productivity, underscoring their sustained impact throughout the period. More recently, Telias I and Zhou J.X entered the field between 2019 and 2020 but demonstrated rapid productivity. The co-authorship network diagram is shown in Figure 4B. Laurent Brochard has the broadest range of collaborations.

**Table 3:** Top 10 active authors with most documents.

Ranks	Authors	Countries	Articles	Total citations	H-index
1	Brochard L.	Canada	11	704	9
2	Foti G.	Italy	9	326	8
3	Scano G.	Italy	9	228	7
4	Bellani G.	Italy	7	108	5
5	Conti G.	Italy	7	212	6
6	Gorini M.	Italy	7	217	6
7	Telias I.	Canada	7	369	5
8	Villamor J.	Spain	7	221	6
9	Zhou J.X.	China	7	31	3
10	Duranti R.	Italy	6	179	6

H-index, Hirsch index.

### 3.4 Analysis of keywords

**Figure 5:**
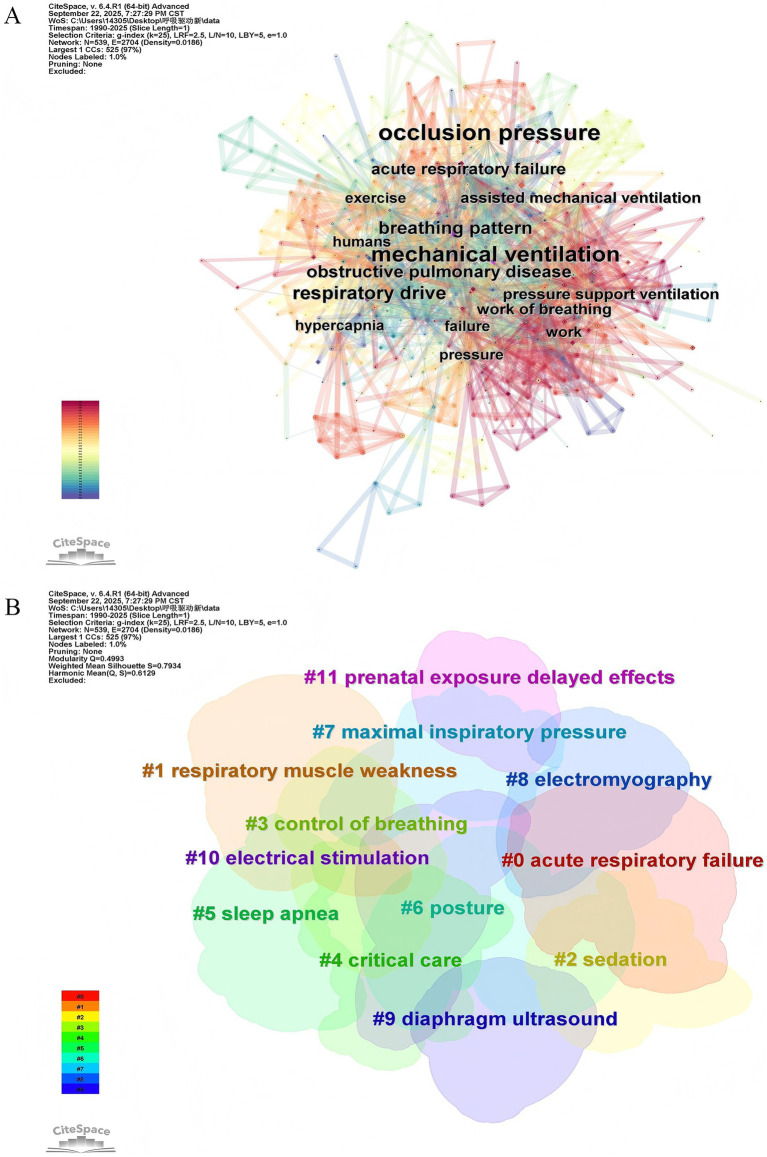
Keywords analysis. **(A)** Co-occurrence network visualization map of keywords. **(B)** Visualization map of keyword clustering module analysis.

**Figure 6:**
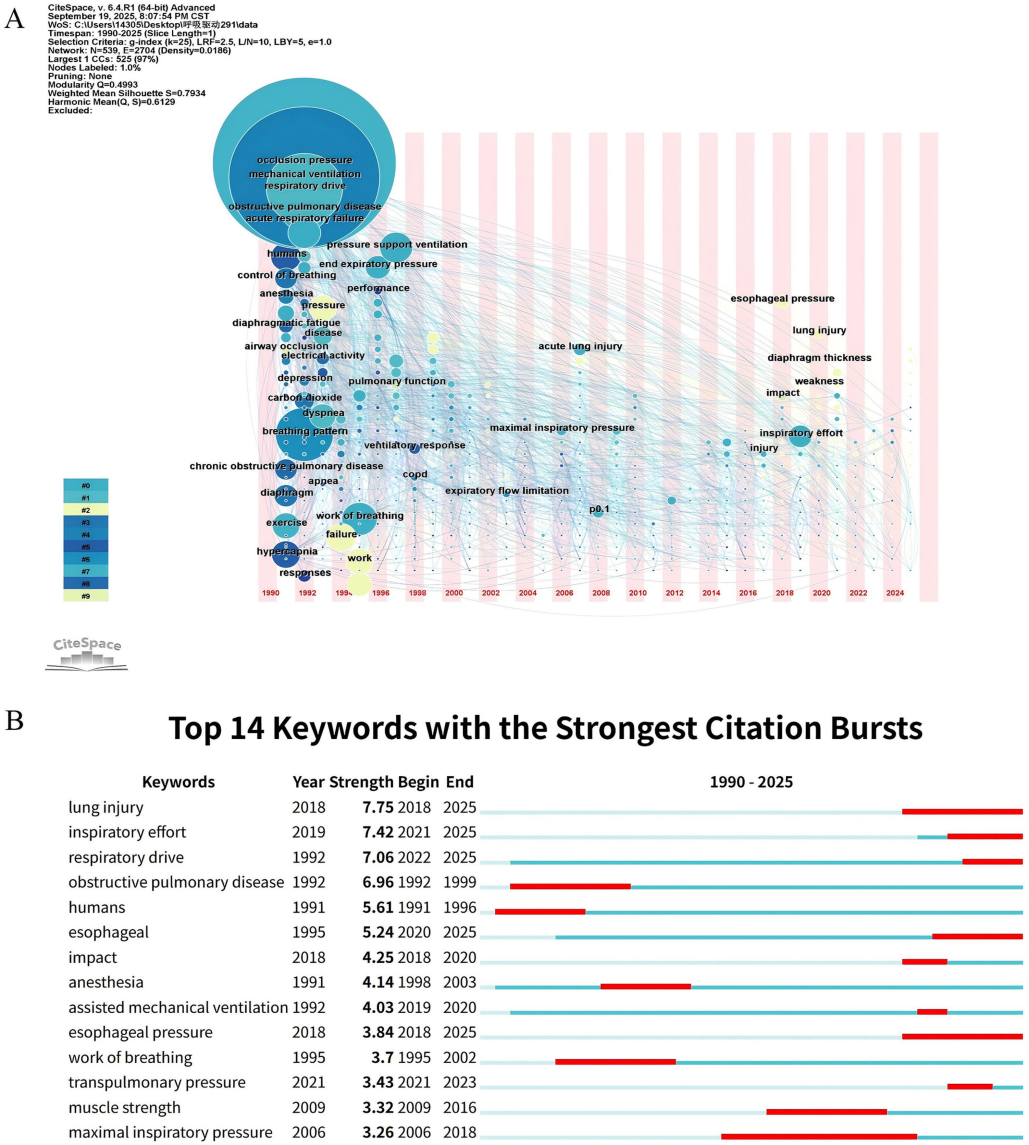
Keywords analysis. **(A)** Timeline graph of keywords. **(B)** Visualization of burst keywords.

A total of 539 keywords were identified, of which 10 occurred more than 20 times. Table 4 lists the top 10 most frequently occurring keywords, with “occlusion pressure” leading at 139 occurrences, followed by “mechanical ventilation” at 114 and “respiratory drive” at 57, aligning with our research theme. The network density observed in the keyword co-occurrence maps was 0.0186, which is generally considered low, indicating that the literature encompasses a broad range of topics (Figure 5A). Twelve clusters were generated using the log-likelihood ratio algorithm to index keyword terms, including diverse themes such as #0 Acute respiratory failure, #1 Respiratory muscle weakness, #2 Sedation, #3 Control of breathing, #4 Critical care, #5 Sleep apnea, #6 Posture, #7 Maximal inspiratory pressure, #8 Electromyography, #9 Diaphragm ultrasound, #10 Electrical stimulation, and #11 Prenatal exposure delayed effects (Figure 5B; Supplementary Table S2). The average silhouette value of the 12 clusters exceeded 0.6, suggesting a high uniformity and reliable analytical results. Additionally, to examine the temporal characteristics within the research fields represented by each cluster, a keyword timeline graph was constructed (Figure 6A). “Burst keywords” are keywords that have been frequently cited over a defined period (30). Figure 6B displays the top 14 keywords exhibiting the most significant surge in citations since 1990. The red bars represent the emergence and persistence of research hotspots (31), with burst durations range from one to twelve years. Keywords such as “maximal inspiratory pressure” (2006–2018), “exercise” (1992–1998), “esophageal pressure” (2018–2025) and “obstructive pulmonary disease” (1992–1999) received the most prolonged attention. Recently, keywords like “respiratory drive,” “lung injury,” and “transpulmonary pressure” have become prominent, indicating that they have received significant attention and may become hotspots in the future. The strongest citation burst was for “lung injure” (7.75), followed by “inspiratory effort” (7.42), “respiratory drive” (7.06), with other keywords having bursts between 3.26 and 6.96.

**Table 4:** The top 10 keywords by frequency ranking.

Ranks	Keyword	Frequency
1	occlusion pressure	139
2	mechanical ventilation	114
3	respiratory drive	57
4	breathing pattern	43
5	obstructive pulmonary disease	35
6	acute respiratory failure	29
7	work of breathing	26
8	assisted mechanical ventilation	24
9	pressure support ventilation	24
10	humans	22

### 3.5 Analysis of reference co-citation

**Figure 7:**
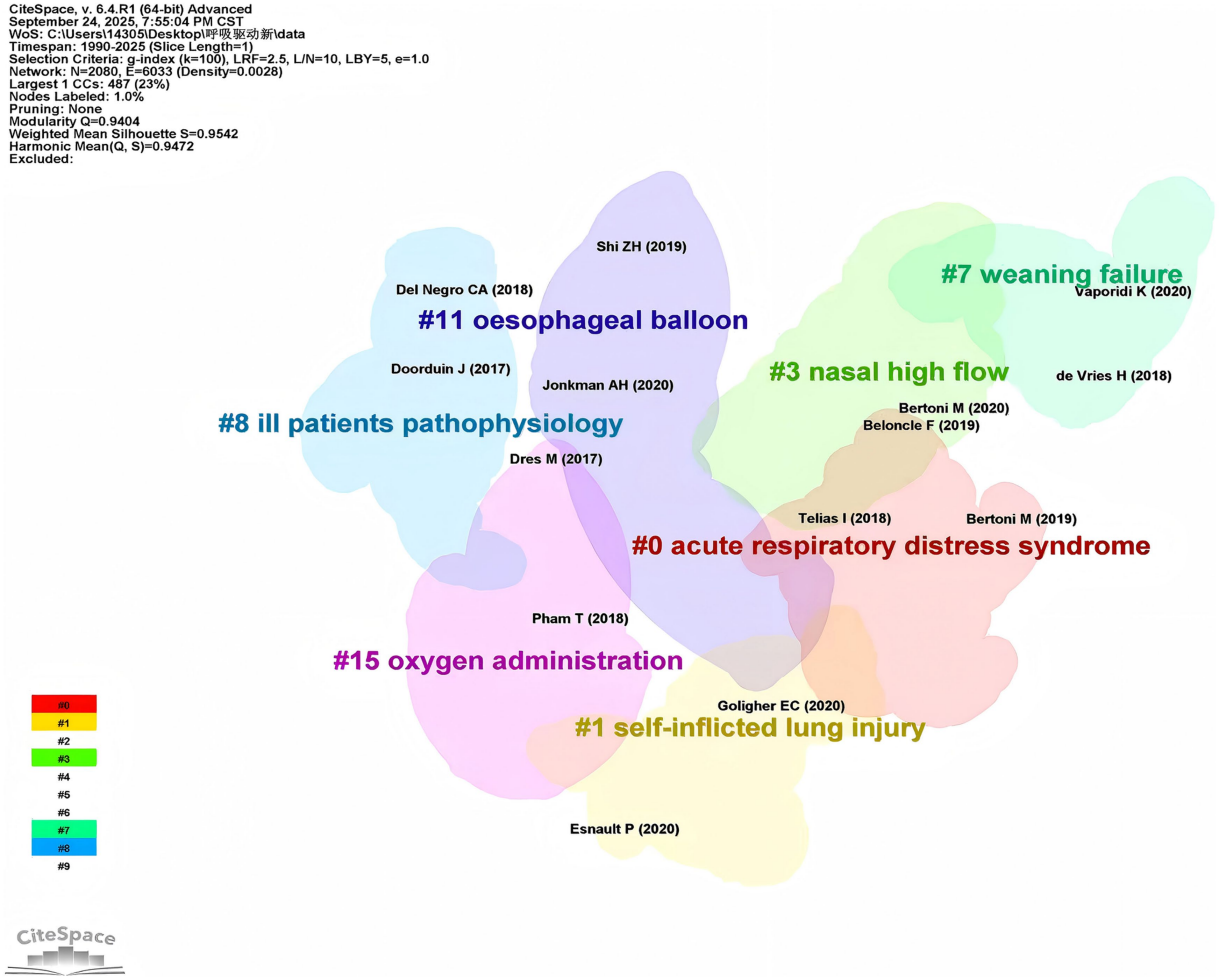
Visualization map of reference co-citation clustering module analysis.

Figure 7 presents a cluster visualization of the reference co-citation network analyzed by CiteSpace. The log-likelihood ratio test was applied for cluster labeling. The analysis resulted in a modularity Q of 0.940, indicating clear cluster separation, and a silhouette value of 0.947, demonstrating excellent clustering homogeneity.

Cluster #0 (Acute respiratory distress syndrome, ARDS) was the largest cluster with 135 members and a high silhouette value of 0.942 (Table 5), indicating strong internal consistency. This cluster focuses on respiratory monitoring and physiology in ARDS. Key citing articles include reviews by Coudroy R et al. (32) and Silva P.L. et al. (33) on respiratory mechanics, as well as studies by Vaporidi K et al. (34) and Telias I et al. (23) on respiratory drive and effort monitoring. Highly cited references within this cluster include Bertoni M et al. [(21), 21 citations] and Telias I et al. [(35), 17 citations], which underscore the relevance of inspiratory effort assessment in ARDS management. Cluster #1 (Self-inflicted lung injury) includes 70 members (silhouette = 0.961, Table 5) and concentrates on inspiratory effort and drive in the context of patient-self -inflicted lung injury. Important citing works by Tonelli R et al. (18) and Jonkman A.H. et al. (36) highlight bedside monitoring techniques. The cluster also contains influential publications such as Goligher E.C. et al. [(37), 18 citations] and Esnault P et al. [(12), 15 citations], emphasizing the role of respiratory drive measurement in preventing lung and diaphragm injury. Cluster #3 (Nasal high flow) comprises 61 members (silhouette = 0.943, Table 5) and highlights the application of high-flow oxygen therapy and its impact on breathing effort. Citing articles by Telias I et al. (23) and Docci M et al. (22) discuss the evaluation of breathing patterns and patient effort under different support conditions. Highly cited papers such as Bertoni M et al. [(38), 17 citations] and Goligher E.C. et al. [(10), 14 citations] appear in this cluster, linking high-flow therapy to respiratory drive monitoring. Cluster #11 (Esophageal balloon) contains 40 members (silhouette = 0.951, Table 5) and emphasizes advanced respiratory monitoring using esophageal pressure measurements. Key citing articles focusing on practical aspects of monitoring inspiratory effort (38, 39).

**Table 5:** The largest 10 reference co-citation clusters.

Cluster ID	Size	Silhouette	Label (LLR)	Average year
0	135	0.942	Acute respiratory distress syndrome	2017
1	70	0.961	Self-inflicted lung injury	2021
3	61	0.943	Nasal high flow	2020
7	46	0.991	Weaning failure	2015
8	44	0.915	Ill patients pathophysiology	2018
11	40	0.951	Esophageal balloon	2020
15	33	0.945	Oxygen administration	2018
20	22	1	Prolonged mechanical ventilation	2019
21	21	0.989	Bedside	2019
28	15	0.993	Acute brain-injured patient	2017

LLR, log-likelihood ratio.

**Figure 8:**
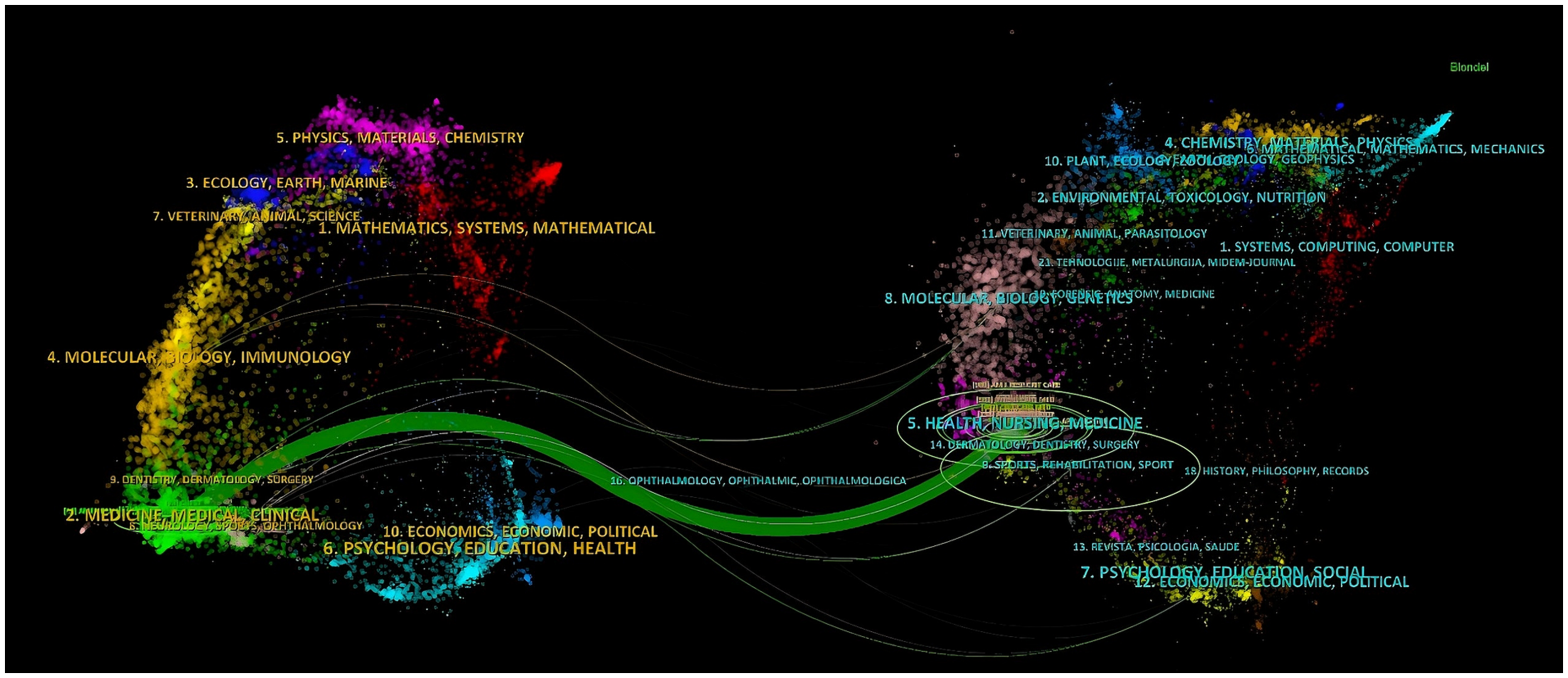
Dual-overlay of the subject spread among the journals. The left cluster represents citing journals, and the right cluster represents cited journals.

Figure 8 presents a dual-overlay created by CiteSpace, illustrating the distribution of subjects among the journals. The dual overlay of the journal maps demonstrates the citation connections between the citing and co-cited journals. The left panel displays a cluster of citing journals, while the right panel shows a cluster of cited journals. The green pathway highlighted in Figure 8 represents the principal citation route, indicating that research articles from medicine, medical, and clinical journals primarily cite works from the health, nursing, and medicine domains.

### 3.6 Analysis of clinical experiments in the PubMed database

A PubMed search covering the period from 1990 to 2025 identified 242 English-language clinical studies. Analysis of these records provided a clinically oriented perspective, complementing the broader bibliometric findings from the WOSCC.

**Figure 9:**
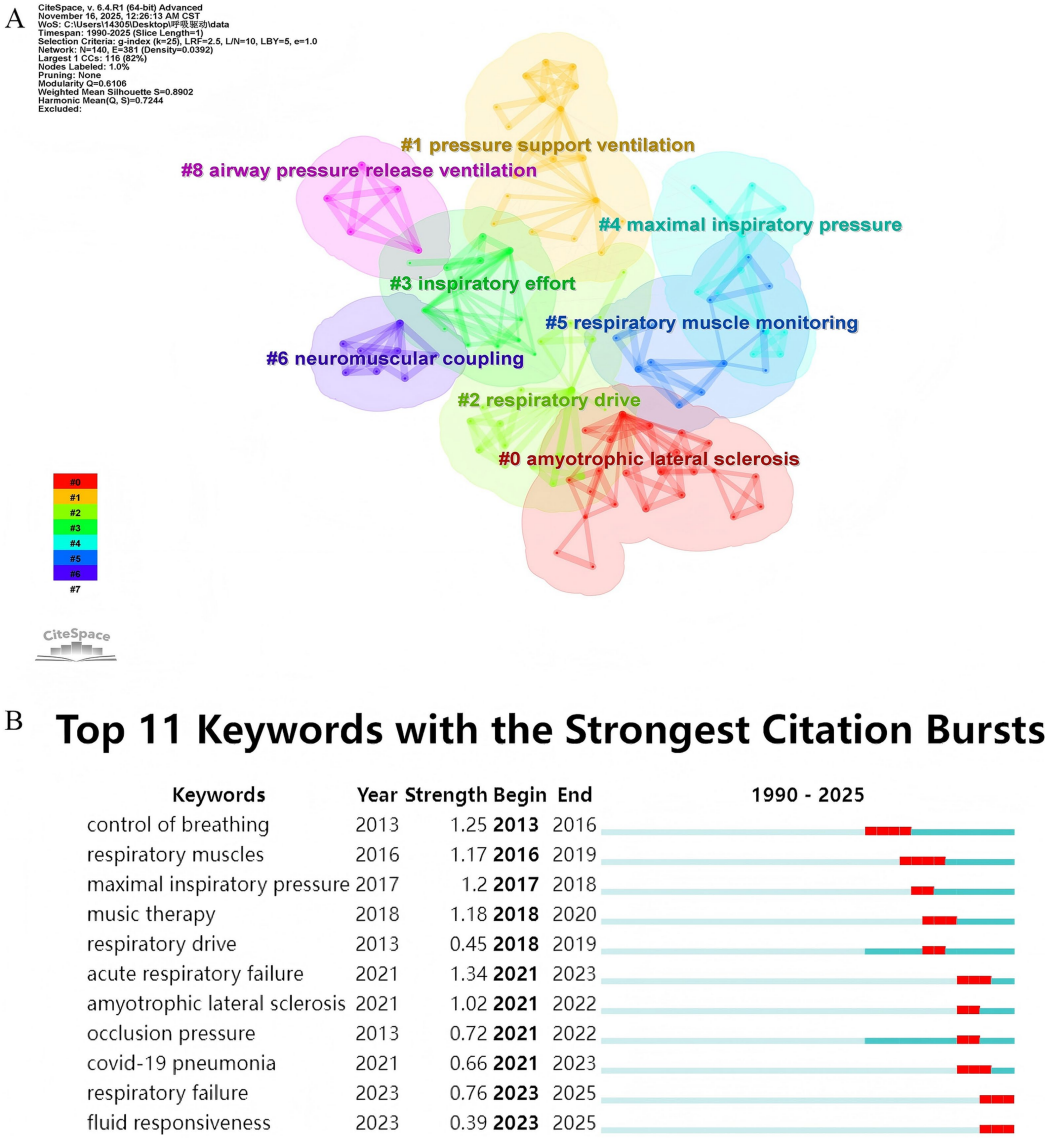
Keywords analysis of PubMed. **(A)** Visualization map of keyword clustering module analysis. **(B)** Visualization of burst keywords.

Keyword co-occurrence clustering of the PubMed dataset revealed distinct research themes (Figure 9A). Major clusters included #0 Amyotrophic lateral sclerosis, #1 Pressure support ventilation, #2 Respiratory drive, #3 Inspiratory effort, #4 Maximal inspiratory pressure and #5 Respiratory muscle monitoring, 6# Neuromuscular coupling, 8# Airway pressure release ventilation. This clustering highlights the clinical research focus on specific ventilation modes, quantitative assessment of muscle strength, monitoring techniques, the neurophysiological control of breathing, and application in specific neuromuscular diseases.

Burst detection analysis identified keywords with the most significant citation bursts, indicating emerging research interests within the clinical literature (Figure 9B). Recent strong bursts were observed for keywords such as “Acute respiratory failure” (Strength: 1.34, 2021–2023), “Amyotrophic lateral sclerosis” (Strength: 1.02, 2021–2022), “COVID-19 Pneumonia” (Strength: 0.66, 2021–2023), “Respiratory failure” (Strength: 0.76, 2023–2025), and “Fluid responsiveness” (Strength: 0.39, 2023–2025). This pattern underscores growing clinical attention towards monitoring inspiratory effort and drive in the context of prevalent critical conditions like severe viral pneumonias and amyotrophic lateral sclerosis, as well as its potential role in guiding fluid management.

## 4 Discussion

This bibliometric analysis provides a comprehensive overview of research trends related to airway pressure-based indexes for monitoring inspiratory effort over the past 35 years. The present study has mapped the intellectual structure, key factors and evolution of this field. The findings reveal a transition from foundational physiology to a crucial theme in contemporary critical care, emphasizing the optimization of ventilator management to protect both the lungs and the diaphragm.

The temporal analysis of publication output shows a near U-shaped trend. The initial high productivity during the early 1990s likely corresponds to the early validation and exploration of occlusion pressure. The following decline may reflect a period where research interests shifted towards other aspects of mechanical ventilation or faced technical challenges associated with occlusion pressure measurements. The significant resurgence after 2011, with annual outputs surpassing earlier peaks, underscores a renewed and growing interest. This change seems driven by the critical need for practical, non-invasive bedside tools to quantify inspiratory effort, especially in the context of preventing ventilator-induced lung injury and diaphragmatic dysfunction (40, 41). The citation peaks in 1999 and 2020 highlight publications that had substantial impact, reflecting key turning points in this field’s development.

The analysis of countries, institutions, and authors reveals a collaborative global research network. Leading contributions from Italy, the USA and France highlight the strong international partnerships in this field. The work of prominent authors such as Brochard L. and Goligher E.C. has been pivotal in applying physiological discoveries to clinical practice. The fact that the top publishing journals (e.g., American Journal of Respiratory and Critical Care Medicine, Chest) are high-impact journals indicates that the researches on airway pressure-based indexes are fully integrated into mainstream intensive care medicine and underscores its clinical relevance.

The keyword clustering analysis reveals the primary research themes and topics in this field. Clusters such as #0 Acute Respiratory Failure, #1 Respiratory Muscle Weakness, #2 Sedation, and #9 Diaphragm Ultrasound exhibit high silhouette scores, indicating clear internal consistency and suggesting that researches cover a broad range of significant clinical and physiological areas. The prominence of Cluster #0 and Cluster #2 underscores the key application of airway pressure-based indexes in managing critically ill patients who are breathing spontaneously, where balancing ventilator support with sedation is essential. These indexes provide objective guidance for titrating sedation to maintain appropriate inspiratory effort (42), thereby facilitating lung-protective ventilation in spontaneously breathing patients. Meanwhile, Cluster #1 reflects sustained scholarly attention toward assessing the strength and function of respiratory muscles, which is particularly relevant for identifying patients at risk of weaning failure and for guiding targeted rehabilitation strategies (43). The continued focus on this cluster emphasizes the clinical importance of quantifying respiratory muscle function to optimize patient outcomes during mechanical ventilation liberation. The distinct formation of Cluster #9 indicates a trend toward integrating different monitoring methods, using pressure-based measures alongside ultrasound imaging to provide a comprehensive assessment of diaphragm health (1). Collectively, these clusters outline the main directions of current researches.

Changes in research focus over time, as indicated by the keyword timeline and burst detection, are strongly corroborated by the reference co-citation analysis. The recent emphasis on keywords such as “respiratory drive,” “lung injury,” and “critically ill patients” highlights current research priorities. This trend is prominently visible in the reference co-citation network, where Cluster #0 (Acute respiratory distress syndrome) and Cluster #1 (Self-inflicted lung injury) emerge as major areas of knowledge. These clusters contain highly cited studies that provide robust evidence for the link between inspiratory effort and ventilator-related lung injury. Additionally, Cluster #3 (Nasal high flow) indicates that the study of airway pressure-based indexes has expanded to include non-invasive breathing support, with research examining the impact of such therapies on the inspiratory effort. The presence of Cluster #11 (Esophageal balloon) further confirms that invasive monitoring of esophageal pressure remains an important reference method for validating the accuracy of non-invasive indices. Thus, the co-citation network not only affirms the current research focus but also illustrates the diversity of clinical and methodological contexts in which this research occurs.

The analysis of the PubMed database not only validates the broad trends identified in the WOSCC but also provides a clearer understanding of the evolving priorities in clinical research. The keyword burst detection is particularly informative, highlighting a clear trajectory from foundational concepts to complex, integrated care challenges. The emergence of “fluid responsiveness” as a burst keyword is significant, suggesting an expanding research frontier that connects respiratory effort assessment with circulatory management in critically ill patients. Simultaneously, the pronounced focus on “Amyotrophic lateral sclerosis” in both clustering and burst analyses underscores the crucial application of these indices in managing progressive neuromuscular disorders, where monitoring respiratory drive and muscle function is vital for prognostic evaluation and guidance in ventilatory support. This focus indicates that the field is maturing beyond general critical care toward a more comprehensive and precisely targeted approach, addressing the complex interplay between the lungs and cardiovascular system in acutely ill patients, as well as the specific needs of neurological populations where respiratory failure is a primary concern.

The airway pressure-based indexes P0.1, ΔPocc, and PMI serve distinct yet complementary roles in monitoring inspiratory effort. P0.1 reflects the intensity of the motor output from the brainstem’s respiratory centers, but its accuracy in assessing inspiratory effort can be affected in patients with neuromuscular dysfunction or abnormal respiratory mechanics, limiting its reliability as a standalone measure in these cases (18). A recent study have indicated that P0.1 had a poor correlation with esophageal pressure swing, work of breathing, and pressure–time product (44). These findings emphasize the need for caution when using P0.1 as a surrogate for inspiratory effort. ΔPocc correlates with total respiratory muscle pressure and lung stress, demonstrating high accuracy in detecting high diaphragm effort (18, 45). Similarly, PMI correlates with the elastic effort measured with esophageal manometry and surface electromyography, proving particularly valuable for detecting over-assistance during mechanical ventilation support (46). Collectively, these indices facilitate a comprehensive assessment from central drive to muscular function, enabling individualized ventilator management when interpreted within the broader clinical context.

Several landmark publications have significantly influenced the field by validating clinical tools and conceptual frameworks. Telias et al. (35) revitalized clinical interest in P0.1, elucidating its role as a measure of respiratory drive and its utility in guiding ventilator support during spontaneous breathing. Subsequently, Bertoni et al. (21) introduced ΔPocc as a novel, non-invasive screening tool, demonstrating its high accuracy in detecting excessive respiratory muscle pressure and transpulmonary driving pressure, both of which frequently arise during assisted ventilation. The comparative performance of these indices was rigorously assessed by de Vries et al. (45), whose research confirmed that both P0.1 and ΔPocc can identify extremes of lung stress and diaphragm effort, with ΔPocc exhibiting superior performance in detecting heightened diaphragmatic effort. In this context, the study conducted by Yang et al. (24) provided pivotal evidence for the diagnostic accuracy of the PMI, establishing it as a dependable non-invasive indicator for assessing inspiratory effort. Their research defined a clinically actionable threshold (e.g., PMI ≤ 0 cmH_2_O to rule out low effort) and demonstrated that PMI could be measured with reasonable reliability at the bedside, thereby enhancing the methods for personalized ventilation strategies aimed at lung and diaphragm protection (24). Concurrently, Soundoulounaki et al. (47) provided crucial methodological insights, revealing that expiratory muscle activity is common and may complicate the interpretation of airway pressure plateaus during spontaneous breathing, thus highlighting the necessity for cautious clinical application. Collectively, these studies underscore a significant shift towards lung- and diaphragm-protective ventilation, aiming to maintain inspiratory effort within a physiological safety range, thereby preventing P-SILI and insufficient effort that results in diaphragm atrophy. The evolution of non-invasive indices such as P0.1 and ΔPocc offers practical tools essential for implementing this paradigm, enabling clinicians to screen for injurious breathing patterns without a routine dependence on invasive monitoring and facilitating timely interventions, such as adjusting support levels or sedation. This personalized approach to ventilation moves beyond generic lung-protective settings to strategies that simultaneously safeguard lung function and preserve respiratory muscle integrity, a concept strongly emphasized in recent guidelines and consensus documents (10).

This study has several limitations that warrant attention. First, the exclusive reliance on the WOSCC as a single data source represents a potential limitation, as it may have resulted in the omission of relevant studies from other databases (e.g., Scopus) or non-English journals, introducing a risk of selection bias (48). While the inclusion of PubMed data enhances the clinical perspective of our analysis, it is important to note that cross-database deduplication was not conducted. Consequently, some publications indexed in both the WOSCC and PubMed may have been counted twice, potentially inflating certain bibliometric indicators and introducing a source of bias in our results. Second, based on temporal bias in citation-based metrics, older publications inherently have a higher cumulative citation count, which may not reflect the true impact of newer work. Third, bibliometrics describes publication trends and relationships but does not does not necessarily imply causation or the true significance of the identified themes. Finally, the search strategy, though comprehensive, might not have captured every relevant publication due to variations in keywords.

## 5 Conclusion

This bibliometric analysis outlines the research status of airway pressure-based indexes for monitoring inspiratory effort. The findings indicate that these indexes are now well-established tools for protecting the lungs and diaphragm during mechanical ventilation. The field is characterized by strong international collaboration and focuses on key areas, including acute respiratory failure and lung injury. Emerging clinical research links respiratory effort to fluid management and expands applications to neuromuscular diseases. These results underscore the clinical significance of this field and support future studies on personalized ventilation strategies.

## Data Availability

The raw data supporting the conclusions of this article will be made available by the authors, without undue reservation.
