# Supplementary material for: Noninvasive monitoring of inspiratory effort in mechanical ventilation: a dual-database bibliometric analysis from 1990 to 2025
Source: Front Med (Lausanne). 2026 Jan 12;12:1747437. doi: 10.3389/fmed.2025.1747437 (PMC12832877; doi:10.3389/fmed.2025.1747437)
Supplement: Supplementary file 1 [file Table_1.DOCX]

Supplementary Material

1. **MATERIALS AND METHODS**

Publications searched in Pubmed database with specific strategy: ( ("P0.1"[Title/Abstract] OR "P100"[Title/Abstract] OR "P-0.1"[Title/Abstract] OR "P(0.1)"[Title/Abstract] OR "occlusion pressure"[Title/Abstract] OR "occlusion pressures"[Title/Abstract] OR "PMI"[Title/Abstract] OR "pressure muscle index"[Title/Abstract] OR "POCC"[Title/Abstract]) AND ( ( "inspiratory effort"[Title/Abstract] OR "inspiratory efforts"[Title/Abstract] OR "breathing effort"[Title/Abstract] OR "breathing efforts"[Title/Abstract] OR "breath effort"[Title/Abstract] OR "respiratory effort"[Title/Abstract] OR "respiratory efforts"[Title/Abstract] OR "respiratory muscle effort"[Title/Abstract] OR "respiratory muscle efforts"[Title/Abstract] OR "inspiratory muscle effort"[Title/Abstract] OR "inspiratory reserve volume"[Title/Abstract] OR "inspiratory reserve volumes"[Title/Abstract] OR "work of breathing"[Title/Abstract] OR "WOB"[Title/Abstract] OR "breathing work"[Title/Abstract] OR "inspiratory work"[Title/Abstract] OR "respiratory work"[Title/Abstract] OR "respiratory capacity"[Title/Abstract] OR "inspiratory capacity"[Title/Abstract] OR "neuromuscular drive"[Title/Abstract] OR "respiratory drive"[Title/Abstract] OR "respiratory drives"[Title/Abstract] OR "inspiratory drive"[Title/Abstract] OR "inspiratory drives"[Title/Abstract] OR "respiration drive"[Title/Abstract] OR "breathing drive"[Title/Abstract] OR "breathing drives"[Title/Abstract] ) OR "Work of Breathing"[Mesh] ) AND English[LA]

# Supplementary Tables

**Table S1 The top 10 most highly cited publications**

| Ranks | Paper | Authors | Years | Journal | Total Citations | TC per Year |
| --- | --- | --- | --- | --- | --- | --- |
| 1 | Changes in the work of breathing induced by tracheotomy in ventilator-dependent patients | Diehl J. L et al. | 1999 | American Journal of Respiratory and Critical Care Medicine | 208 | 7.70 |
| 2 | Patterns and predictors of sleep disordered breathing in primary myopathies | Ragette R et al. | 2002 | Thorax | 166 | 6.92 |
| 3 | Respiratory Drive in Critically Ill Patients. Pathophysiology and Clinical Implications | Vaporidi K et al. | 2020 | American Journal of Respiratory and Critical Care Medicine | 165 | 27.50 |
| 4 | Bedside waveforms interpretation as a tool to identify patient-ventilator asynchronies | Georgopoulos D et al. | 2006 | Intensive Care Medicine | 144 | 7.20 |
| 5 | Ventilatory drive and respiratory muscle function in pregnancy | Contreras G et al. | 1991 | The American Review of Respiratory Disease | 135 | 3.86 |
| 6 | Airway Occlusion Pressure as an Estimate of Respiratory Drive and Inspiratory Effort during Assisted Ventilation | Telias I et al. | 2020 | American Journal of Respiratory and Critical Care Medicine | 129 | 21.50 |
| 7 | Physiologic determinants of ventilator dependence in long-term mechanically ventilated patients | Purro A et al. | 2000 | American Journal of Respiratory and Critical Care Medicine | 126 | 4.85 |
| 8 | Effects of obesity on breathing pattern, ventilatory neural drive and mechanics | Chlif M et al. | 2009 | Respiratory physiology & neurobiology | 115 | 6.76 |
| 9 | Hyperleptinaemia, respiratory drive and hypercapnic response in obese patients Assisted Ventilation | Campo A et al. | 2007 | The European respiratory journal | 105 | 5.53 |
| 10 | P0.1 is a useful parameter in setting the level of pressure support ventilation | Alberti A et al. | 1995 | Intensive Care Medicine | 96 | 3.10 |

**Table S2 Keyword clustering module analysis**

| Number | Size | Silhouette | Name | Keyword |
| --- | --- | --- | --- | --- |
| #0 | 107 | 0.666 | Acute respiratory failure | acute respiratory failure; pressure support ventilation; positive airway pressure; obstructive pulmonary disease; end expiratory pressure |
| #1 | 74 | 0.735 | Respiratory muscle weakness | respiratory muscle weakness; respiratory muscle strength; dyspnea; mechanical ventilation; disease |
| #2 | 53 | 0.843 | Sedation | sedation; acute respiratory distress syndrome; respiratory effort; esophageal pressure; ultrasound |
| #3 | 44 | 0.857 | Control of breathing | control of breathing; quadriplegia; CO_2_ response ventilation; laryngeal chemoreflex apnea; CO_2_ response |
| #4 | 41 | 0.773 | Critical care | critical care; isoflurane; calves; CO_2_ rebreathing; depression |
| #5 | 40 | 0.825 | Sleep apnea | sleep apnea; inspiratory impedance; carotid body; chemosensitivity; chemoreceptors |
| #6 | 40 | 0.781 | Posture | posture; minute ventilation; respiratory failure; hypercapnic stimulus; neuromuscular blockade |
| #7 | 36 | 0.902 | Maximal inspiratory pressure | maximal inspiratory pressure; airway resistance; acute intermittent hypoxia; amyotrophic lateral sclerosis; occlusion pressure |
| #8 | 36 | 0.882 | Electromyography | electromyography; chronic obstructive pulmonary disease; mouth occlusion pressure; broxaterol; chemoresponsiveness |
| #9 | 21 | 0.913 | Diaphragm ultrasound | diaphragm ultrasound; resistive loads; oxygen therapy; interoceptive conditioning; post-arrest |
| #10 | 20 | 0.846 | Electrical stimulation | electrical stimulation; upper airways; sniff; perceived leg exertion; hypoxic apnea |
| #11 | 13 | 0.946 | Prenatal exposure delayed effects | prenatal exposure delayed effects; smoking water pipes; voluntary contraction; carbon monoxide; osteoporosis |
